# A Direct Method for Determining Toricity Ratios of Toric Intraocular Lens Calculators

**DOI:** 10.1038/s41598-018-22591-4

**Published:** 2018-03-15

**Authors:** Lauren G. Gabra, Samir I. Sayegh

**Affiliations:** The Eye Center, Champaign, IL 61820 United States

## Abstract

This study develops a method to determine toricity ratios used by arbitrary toric intraocular lens calculators. Access to this information allows for the improvement of refractive results. We derive the Sayegh-Gabra formula, which uses input and output parameters in a toric calculator to extract toricity ratios that are typically not disclosed. We illustrate the method on a number of commercial calculators. For each calculator, high, average, and low axial length values are crossed with high, average, and low mean corneal power values to generate a 3 × 3 matrix. A toricity ratio is generated for each axial length and mean corneal power pair. We thus identify several toric lens manufacturers’ calculators that use a constant toricity ratio, often 1.46. Some others use a variable ratio centered at 1.46, but varying as axial length and mean K increases over a range of values corresponding to physiological myopia and hyperopia. There is an emerging trend away from constant toricity ratios. Using our methodology, it is possible to extract the toricity ratio used by specific calculators/manufacturers, distinguish those using constant versus variable toricity ratios, and use this information to improve surgical outcomes by refining current and future toric intraocular lens calculators.

## Introduction

Though toric intraocular lenses (tIOL) are relatively new in the armamentarium of the cataract refractive surgeon, the efficacy of tIOLs benefiting patients with cataracts, refractive errors, and corneal astigmatism has been well established^1–3^. Considering 20% to 30% of patients who have cataract surgery have corneal astigmatism of 1.25 diopters (D) or higher, correction at the time of cataract surgery, leading to emmetropia or to a pre-specified target refraction, has become paramount^1,4,5^. It is essential to optimize surgical outcomes by considering the relationship between the intraocular lens (IOL) and corneal toricities, variability of which is impacted by the distance between the IOL and corneal planes, or effective lens position (ELP)^6^. To achieve or approach spectacle independence and alleviate patients’ financial burden, it is paramount calculators suggest lenses that will yield optimal refractive results.

The purpse of this study is to describe a method to compute the toricity ratio used by specific toric calculators, and its dependence on axial length and mean corneal power, using specific evaluations performed on the native calculators. *Toricity ratio*, *τ*, *is the ratio of the toricity or cylinder of the IOL at the IOL plane to the equivalent toricity at the corneal plane*. It is the factor that transforms IOL plane toricity provided by the manufacturer into an equivalent corneal plane toricity, needed for the correction of the patient measured corneal astigmatism^7^. We derive the Sayegh-Gabra formula (see Methods), an explicit expression for τ, and apply it to distinguish calculators using a constant toricity ratio from those using a variable ratio, which are explicitly computed. These values are presented in illustrative 3 × 3 squares that may hold a key to improved outcomes for patients undergoing implantation of toric intraocular lenses.

tIOL calculators are produced by several manufacturers, with many utilizing a constant toricity ratio^7,8^. Considering such calculators do not compute the ELP, the influence of shallower or deeper eyes and other biometric parameters on the toricity ratio is disregarded. This use of a fixed toricity ratio implies the calculation is made based on “average” rather than “customized” parameters, a limitation that may lead to unexpected refractive errors and have implications on surgical outcomes^8,9^. For example, Savini, G. *et al*. have identified that a toricity ratio <1.41 led to overcorrection of astigmatism relative to the value reported by the manufacturer’s online calculator, while a toricity ratio >1.60 led to under correction of the astigmatism^7^.

## Results

Manufacturer calculators evaluated included those for the original Alcon, Abbott (now Johnson and Johnson), and Bausch and Lomb, for tIOLs available worldwide including the United States, and Acriva BB Toric, HOYA Toric IOL and PhysIOL Ankoris and FineVision Toric IOLs as illustrative examples of tIOLs available outside of the United States. Alcon Acrysof Toric IOL, Abbott Tecnis Toric IOL, and Bausch and Lomb enVista and Trulign Toric IOL toric lenses are all FDA approved. One of the first results we established is that the toricity ratios for all calculators, for the full range of values examined, did not depend on the individual values of K steep and K flat, but on their mean value.

Calculators found to use a fixed toricity ratio for their tIOLs included the VSY Biotechnology BV Acriva^UD^ Toric calculator, HOYA Toric calculator, and the original Alcon AcrySof tIOL Calculator (Table 1), all using a ratio centered at 1.46.

**Table 1:** A table illustrating the toricity trend utilized by the Alcon for Acrysof Toric IOL calculator. Mean K values of 39 D, 43 D, and 47 D and axial length values of 21 mm, 24 mm, and 27 mm are paired. The toricity ratio resulting from each pair is displayed in the corresponding box. No statistical data analysis was required.

	Original Alcon Acrysof Calculator Toricity Trend			
47		1.46	1.46	1.46
44		1.46	1.46	1.46
39		1.46	1.46	1.46
Mean K (D)				
	Axial Length (mm)	21	24	27

The original PhysIOL for Ankoris and FineVision calculators also utilized a constant toricity ratio of 1.46 as of June 2017. However, as of July 2017, PhysIOL has updated both calculators and preliminary review has shown utilization of a variable toricity ratio. Only the updated calculators are currently available and accessible.

Preliminary review of the updated Alcon calculator, also implemented in 2017, indicates a similar methodology of utilizing a variable toricity ratio has finally been adopted by Alcon. Both the original and updated calculators are available and accessible.

The Bausch and Lomb enVista (Table 2) and Trulign calculators were found to use a constant toricity ratio of 1.43 and 1.50, respectively.

**Table 2:** A table illustrating the toricity trend utilized by the Bausch and Lomb for enVista Toric IOL calculator. Mean K values of 39 D, 43 D, and 47 D and axial length values of 21 mm, 24 mm, and 27 mm are paired. The toricity ratio resulting from each pair is displayed in the corresponding box. No statistical data analysis was required.

	Bausch and Lomb enVista Calculator Toricity Trend			
47		1.43	1.43	1.43
44		1.43	1.43	1.43
39		1.43	1.43	1.42
Mean K (D)				
	Axial Length (mm)	21	24	27

As shown in Table 3, Abbott Tecnis IOL calculator uses a variable ratio which increases as corneal power and axial length increase, indicating that the calculator is taking both axial length and mean corneal power into account. While the values of axial length and mean corneal power considered were reasonably common, we found that as the trend continues toricities ranging from 1.30 to 1.90 resulted.

**Table 3:** A table illustrating the toricity trend utilized by the Abbott Medical Optics (AMO) for Tecnis Toric IOL calculator. Mean K values of 39 D, 43 D, and 47 D and axial length values of 21 mm, 24 mm, and 27 mm are paired. The toricity ratio resulting from each pair is displayed in the corresponding box. No statistical data analysis was required.

	Abbott Tecnis Calculator Toricity Trend			
47		1.47	1.63	1.75
44		1.40	1.52	1.56
39		1.33	1.38	1.42
Mean K (D)				
	Axial Length (mm)	21	24	27

## Discussion

Emmetropia, or specific desired refractive targets, following cataract surgery can be achieved, so long as careful preoperative measurements and accurate methodologies are utilized. Because of the absence of calculation formula and variables used by the manufacturers for the toric calculation, the analysis of this discrepancy, and patient benefits, may be hindered. The methods presented here demonstrate a possible remedy to this situation.

We applied our general method and extracted toricity ratios for all calculators considered. We then applied the results to specific calculators from manufacturers to establish clinically valuable conclusions. We demonstrated for example that the Abbott Tecnis Toric IOL calculators use a variable toricity ratio which follow the trend demonstrated by Goggin, Savini *et al*., and others, namely that as corneal power and axial length increase, toricity ratio should increase^7,8^. We also demonstrated that the VSY Biotechnology BV Acriva^UD^ Toric calculator, HOYA Toric calculator, the original Alcon AcrySof tIOL Calculator, and the original PhysIOL Ankoris and FineVision Toric IOLs calculators, amongst many others, do not take corneal power and axial length relationships into account during their computations, and demonstrated their use of a common constant toricity ratio centered at 1.46. The limitations of calculators using a constant toricity ratio are further illustrated when considering toricity ratios can be as small or smaller than 1.30 for very short eyes and/or flat K values, and as large or larger than 1.90 for very long eyes and/or steep K values. Potential consequences, including significant uncorrected astigmatism and potential suboptimal choices of toric lenses have been illustrated in recent publications^9,10^.

We also show that the Bausch and Lomb calculator for the Trulign and enVista tIOLs, while using a constant toricity ratio, yielded a value that is somewhat different from other calculators also using constant toricity. This is consistent with what was observed by Dihowm, Hjelmstad, and Sayegh, where different residual astigmatism values were noted for the same toricity tIOL by different manufacturers’ calculators^11^. This may correspond to a different average ELP, a hypothesis we are currently investigating.

The Sayegh-Gabra formula allows surgeons, manufacturers, and even patients, to identify calculators taking biometric differences into consideration by the utilization of a variable toricity ratio. Awareness of which calculators use a constant toricity ratio is vital for surgeons when operating on eyes that are not “average”, i.e. having keratometry and/or axial length values considerably larger or smaller than about 43 D or 24 mm. In such cases, it is important to know what type of tIOL calculator is used for lens selection. An experienced surgeon following years of using constant ratio calculators may be accustomed to modifying lens recommendations. Switching to a calculator implementing a variable toricity ratio, their modification may lead to unintended refractive results. A less experienced surgeon operating on a similar eye may use a calculator with a constant toricity ratio without any corrections, potentially also yielding suboptimal results. It is thus vital that surgeons understand the implications of toricity ratios and test whether a calculator operates using a constant or variable ratio.

We remain optimistic that manufacturers and designers of toric intraocular lens calculators will take into consideration variability amongst patients and use variable ratio techniques to help achieve optimal outcomes. Some toric IOL manufacturers such as Alcon and PhysIOL have already adopted such methodologies.

The simplicity of the Sayegh-Gabra formula lends itself to applications by not only cataract and refractive surgeons, but also by patients with minimal knowledge of toric intraocular lenses. In an era of personalized medicine and high expectations, patients can use their personal measurements to determine if further discussion of lens choice is warranted.

We are currently investigating comparisons of accurate predictions enhanced by our methods to techniques such as minimizing the effect of tIOL rotation or postoperative laser modification of the implant.

By understanding the methods used by different manufacturers, and suggesting improvements in calculation methods, we can optimize the choice of tIOLs for patients undergoing refractive cataract surgery.

## Methods

This study evaluated methods of computing and selecting tIOLs and did not involve patients. Therefore, approval by the local clinical research ethics committee was not required.

We considered toric calculators of the following manufacturers for their respective tIOLs: Abbott Medical Optics (AMO) for Tecnis Toric IOL, VSY Biotechnology BV for Acriva BB Toric, Bausch and Lomb for enVista Hydrophobic Toric Acrylic Intraocular Toric and Trulign Toric, Hoya: Toric IOL, the original Alcon: Acrysof Toric IOL, the new Alcon Toric IOL calculator, the original PhysIOL for Ankoris and FineVision IOL calculators, and the new PhysIOL for Ankoris and FineVision IOL calculators. Calculations were performed July 2014 through July 2017^12^.

For each tIOL calculator considered, we ran computations varying the biometric variables used by the calculator, IOL powers and corneal powers, and recorded input values and values for residual astigmatism. Flat keratometry readings ranged from 38.0 D to 46.0 D at 0 degrees, while the steep keratometry reading ranged from 39.0 D to 52.0 D. Beginning with a difference in steep and flat keratometry values of 1.00 D, the difference in values was incremented in steps of 0.25 D. Calculations were made with steep astigmatism at 90 degrees though it was verified that the axis of steep astigmatism did not affect the result. IOL spherical equivalent (SE) values ranged from 9.0 D to 34.0 D. Surgically induced astigmatism was assumed to be 0 as it has no bearing on the problem of interest. Originally, only the AMO IOL Calculator Platform for Tecnis Toric IOL had axial length as an available or required field.

The IOL sphere power was calculated by inverting the SRK/T formula and determining the IOL sphere that would result in a target of plano for a given axial length and mean K. In constructing our tables for each case, axial length and its corresponding flat and steep K values were input and the resulting expected residual astigmatism and cylinder at the IOL plane were recorded. The Sayegh-Gabra formula, derived in the next section, was then used to determine the toricity ratio.

### Derivation of the Sayegh-Gabra formula for the calculation of toricity ratio

We denote by K_*s*_ and K_*f*_, the steep and flat meridian powers of the cornea, respectively. The difference between them, the absolute value of the magnitude of corneal astigmatism or cylinder is denoted *K*_*c*_:

1 $${K}_{c}={K}_{s}-{K}_{f}$$

and is a positive number. When there is no surgically induced astigmatism (SIA), this value is equivalent to the cross cylinder (*C*_*c*_), given by:

2 $$\overline{{C}_{c}}=\overline{{K}_{c}}+\overline{SIA}$$

3 $$\bar{{C}_{c}}=\bar{|{C}_{c}|}$$

Where a bar represents “vector” notation for astigmatism and the plus sign represents vector addition, and |*C*_*c*_| is the magnitude of *C*_*c*_. *C*_*c*_ is the cross cylinder of the corneal astigmatism and the SIA (and any other intervention altering the corneal astigmatism, such as limbal relaxing incisions (LRIs)). As demonstrated by Chen and Sayegh, most toric calculators compute *C*_*c*_ correctly and display it^13^. The topic of computing the *C*_*c*_ is outside the scope of this article but the method, its popularization and many of its applications, have been well documented throughout the centuries^14–19^.

The toric IOL has a certain toricity or magnitude of astigmatism, *at the IOL plane*, also representing the difference of its power in the steep (L_*s*_) and flat (L_*f*_) meridians, and provided by the lens manufacturers. This positive number is denoted by *L*_*c*_.

*L*_*c*_ has an equivalent value at the corneal plane. This value is given by $\frac{Lc}{\tau }$ where *τ* represents a “toricity ratio” that transforms IOL plane toricity into an equivalent corneal plane toricity.

The residual astigmatism, *r*, after attempting to correct *C*_*c*_ by a toric IOL of toricity *L*_*c*_ is then given by:

4 $$r={C}_{c}-\frac{{L}_{c}}{\tau }$$

A positive value of *r* denotes under-correction of astigmatism by the toric IOL, corresponding to no shift in the axis of the astigmatism. A negative value denotes an over-correction, and is indicated by a 90° shift in the axis of the astigmatism.

Solving (4) for τ, we obtain the Sayegh-Gabra formula:

5 $$\tau =\frac{{L}_{c}}{{C}_{c}-r}\,\,\,\,\,({\rm{Sayegh}}-{\rm{Gabra}})$$

Under the assumption the SIA is equal to 0, the following equation holds:

6 $$\overline{{C}_{c}}=\overline{{K}_{c}}$$

The Sayegh-Gabra formula becomes:

$$\tau =\frac{{L}_{c}}{{K}_{c}-r}\,\,\,\,(5^{\prime} ,{\rm{Sayegh}}-{\rm{Gabra}})$$

and can now be applied without needing to compute *C*_*c*_ values.

**Figure 1:**
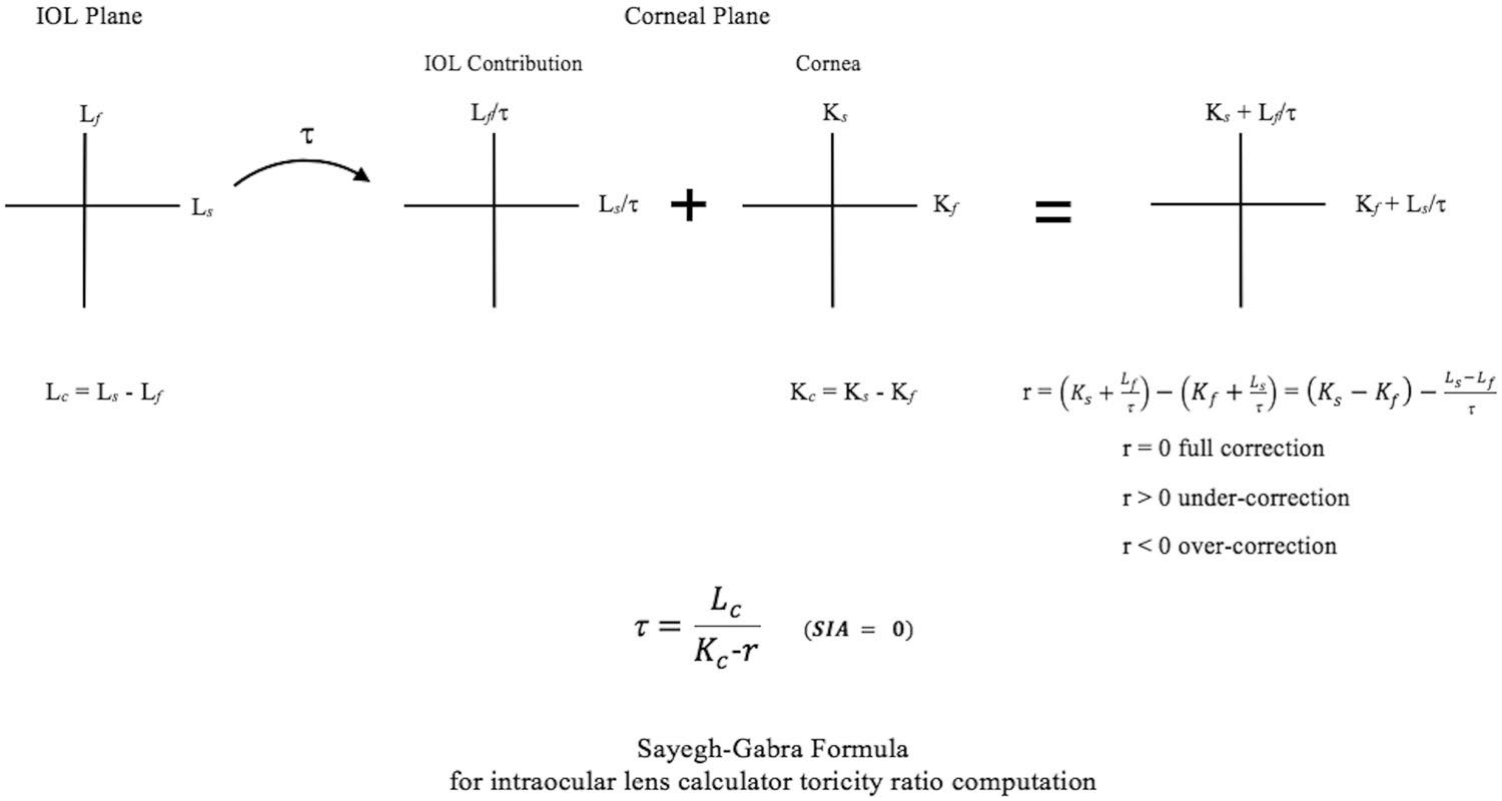
A schematic representation of the Sayegh-Gabra formula derivation, providing an explicit expression for the toricity ratio used by any tIOL calculator.

This expression for τ can be used to investigate the values of toricity ratio used in any toric IOL calculator. This method makes no assumption about, and is independent of, biometric variables used to predict toricity ratio. Figure 1 shows a schematic representation of this derivation.

### Example (original Alcon Acrysof Toric Calculator)

**Figure 2:**
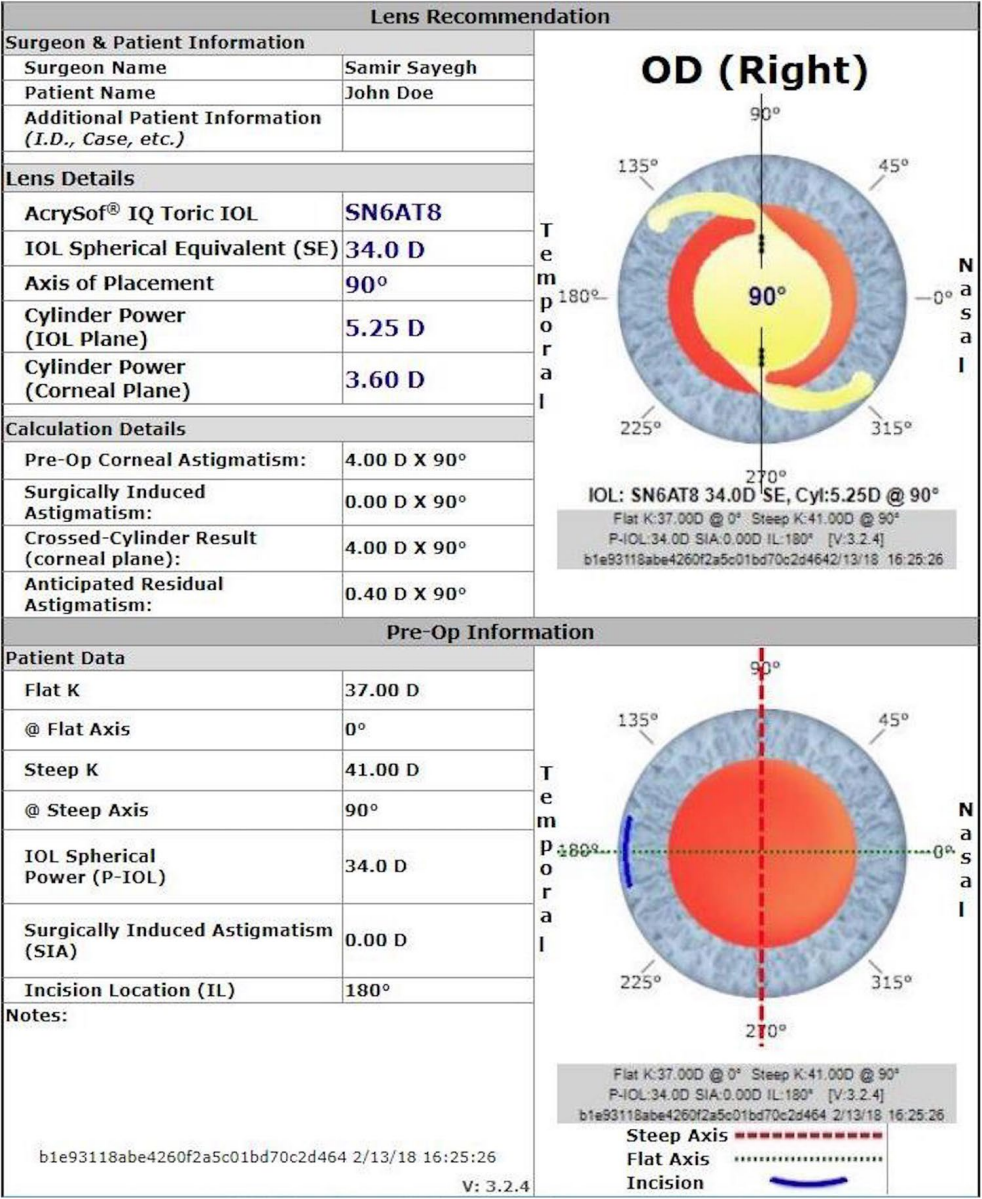
A sample calculation of the Alcon for Acrysof Toric IOL calculator. This calculation displays a mean K of 39.0 D with a sphere power of 34.0 D. The recommended IOL model is the SN6AT8.

A flat K of 37.0 D and steep K of 41.0 D results in a mean K of 39.0 D. Inverting SRK/T, we found a target of plano and axial length of 21.00 mm requires a 34.0 D lens. (Other IOL calculation formulas give a consistent result.) These values were input into the Alcon Acrysof Toric calculator and the SN6AT8 lens was recommended, yielding a residual astigmatism of +0.40 D (Fig. 2).

Axial length was unavailable as an input field. We now apply the Sayegh-Gabra formula. The +0.40 D residual astigmatism value was subtracted from the cross cylinder astigmatic value, 4.00 D, (equal to the corneal astigmatism since SIA is 0), resulting in a computed 3.60 D toricity at the corneal plane. Because the SN6AT8 lens has a power of 5.25 D at the IOL plane, 5.25 was divided by 3.60 resulting in a toricity ratio of 1.46.

### Example (AMO IOL Platform Calculator for Tecnis Toric IOL)

**Figure 3:**
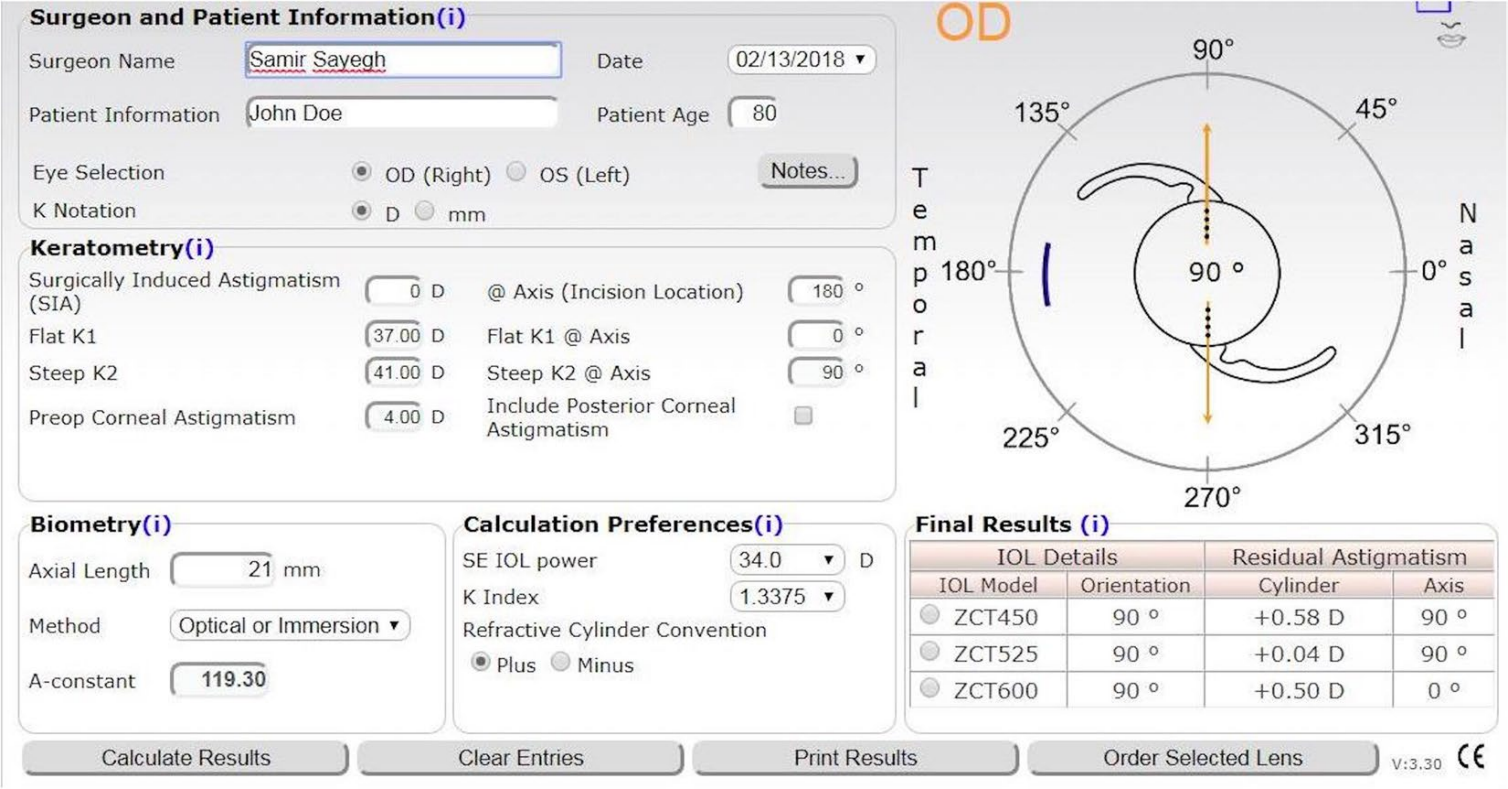
A sample calculation of the Abbott Medical Optics (AMO) for Tecnis Toric IOL calculator. This calculation displays a mean K of 39.0 D and axial length of 21.00 mm with a sphere power of 34.0 D. The recommended IOL models are the ZCT450, ZCT525, and the ZCT600.

Values were repeated above for a mean K of 39.0 D and axial length of 21.00 mm, requiring a 34.0 D lens. These values were input into the Abbott calculator, with zero surgical astigmatism, and three toric lenses were recommended. The first listed lens was the ZCT450, yielding a residual astigmatism of 0.58 D (Fig. 3).

As this is an under-correction, signified by the astigmatic axis remaining at 90°, this residual astigmatism is positive. The Sayegh-Gabra formula was then applied. The +0.58 D residual astigmatism value was subtracted from the cross cylinder astigmatic value, 4.00 D, (equal to the corneal astigmatism since SIA is 0), resulting in a computed 3.42 D toricity at the corneal plane. Because the ZCT450 lens has a power of 4.50 D at the IOL plane, 4.50 was divided by 3.42 resulting in a toricity ratio of 1.32. The ZCT525 was recommended and yielded a residual astigmatism of +0.04. Because the ZCT525 lens has a power of 5.25 D at the IOL plane, 5.25 was divided by 3.96 for a toricity ratio of 1.33. Utilizing the ZCT600 lens, the residual astigmatism yielded 0.50 D. Because this is an overcorrection, signified by the axis alternating to 0°, we take this residual astigmatism to be negative. Subtracted from the 4.00 D cross cylinder, we find the toricity at the corneal plane to be 4.50 D. Because the ZCT600 lens has a power of 6.00 D at the IOL plane, 6.00 was divided by 4.50 resulting in a toricity ratio of 1.33.

### Example (Bausch and Lomb enVista Toric IOL Calculator)

**Figure 4:**
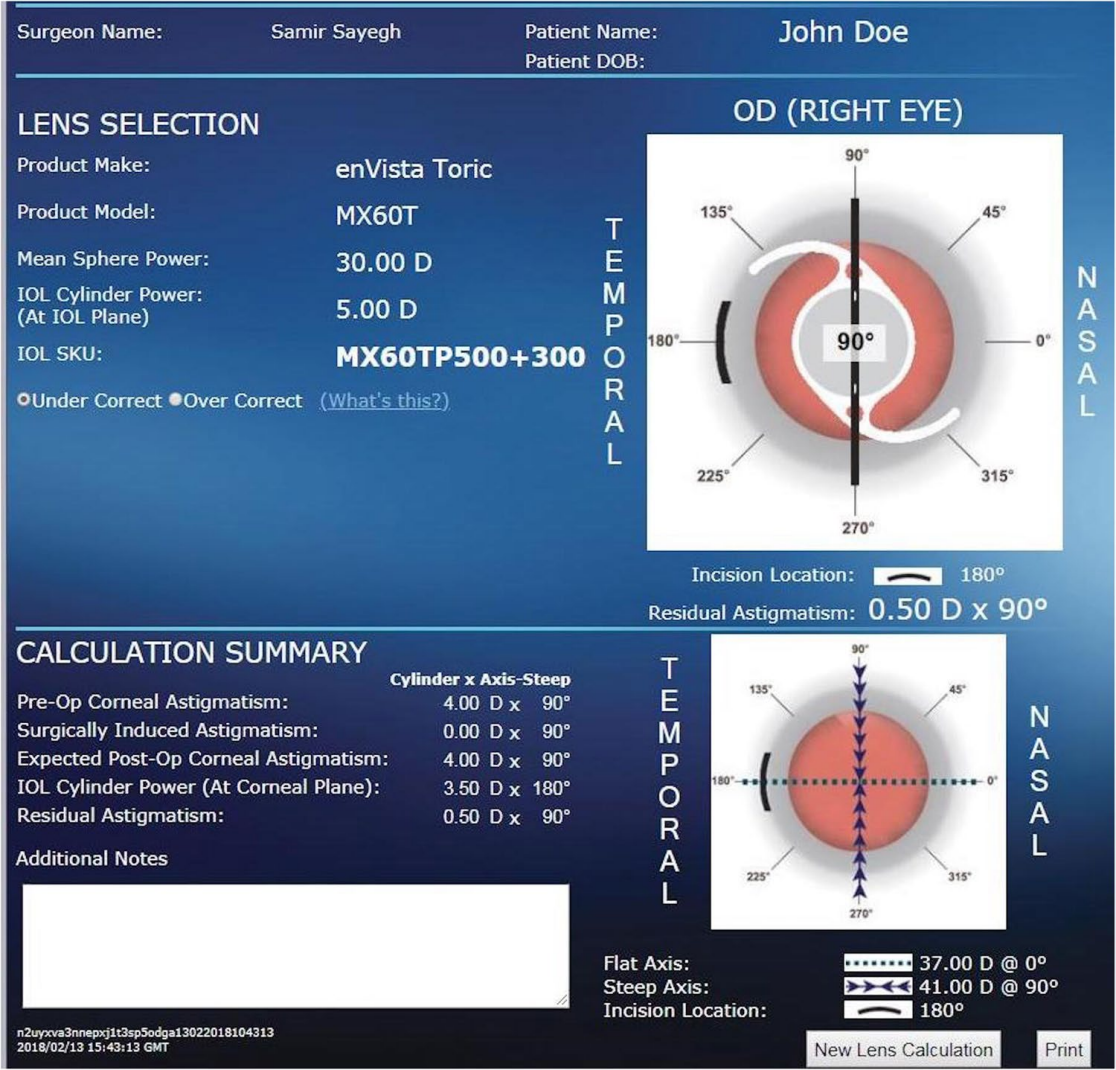
A sample calculation of the Bausch and Lomb for enVista Toric IOL calculator. This calculation displays a mean K of 39.0 D with a sphere power of 30.0 D. The recommended IOL model is the MX60T.

Values were repeated above for a mean K of 39.0 D and axial length of 21.00 mm, requiring a 30.0 D lens. These values were input into the Bausch and Lomb enVista calculator and the MX60T lens was recommended, yielding a residual astigmatism of +0.50 D (Fig. 4).

Axial length was unavailable as an input field. The Sayegh-Gabra formula was then applied, yielding a toricity ratio of 1.43.

Axial length values and mean corneal power value ranges were used to create a two-dimensional matrix which represented the trend of the toricity ratio as a function of these variables for each calculator. On the horizontal axis was increasing axial length, which had three values corresponding to short, average, and long eyes of 21.00 mm, 24.00 mm, and 27.00 mm, respectively. Along the vertical axis was corneal power, which had three values corresponding to low, average, and high mean corneal powers of 39.0 D, 43.0 D, and 47.0 D, respectively. A combination of each axial length with each corneal power produced a 3 × 3 square for a total of 9 ranges of toricity ratio values. If more than one lens was recommended by a toric calculator, the toricity ratio represented by the lens predicting the smallest residual astigmatism was chosen.

### Data availability

The datasets generated during and/or analyzed during the current study are available from the corresponding author on reasonable request.
